# Shortening/re-lengthening and nailing versus bone transport for the treatment of segmental femoral bone defects

**DOI:** 10.1038/s41598-023-40588-6

**Published:** 2023-08-16

**Authors:** Na Yang, Teng Ma, Lu Liu, YiBo Xu, Zhong Li, Kun Zhang, Qian Wang, Qiang Huang

**Affiliations:** Department of Orthopedics, Hong Hui Hospital, Xi’an Jiaotong University, Xi’an, 710054 Shaanxi China

**Keywords:** Trauma, Bone

## Abstract

Segmental femoral bone defects are a severe challenge for orthopedic trauma surgeons. The objective of this study was to compare the efficacy of the shortening/re-lengthening and nailing (SRN) technique with the traditional bone transport (BT) technique in treating patients with such defects. A retrospective study was conducted involving 46 patients with segmental femoral bone defects, with 21 cases treated using the SRN technique (SRN group) and 25 cases managed with the traditional BT technique (BT group). The mean length of the bone defect was 5.8 ± 1.1 cm in the SRN group and 6.1 ± 1.6 cm in the BT group. Various parameters including time in frame, external fixation index, self-rating anxiety scale (SAS) scores, bone healing scores, limb function scores, and complications were recorded. The mean time in frame for the SRN group was 3.7 ± 1.4 months, significantly shorter than the 9.4 ± 3.7 months observed in the BT group (*p* < 0.05). Furthermore, the mean external fixation index for the SRN group was 0.62 ± 0.12 months/cm, significantly lower than the 1.50 ± 0.19 months/cm observed in the BT group (*p* < 0.05). There were no significant differences in bone healing scores between the SRN and BT groups (*p* = 0.237). The SAS scores and incidence of complications were significantly lower in the SRN group compared to the BT group (*p* < 0.05). Overall, the SRN technique demonstrated superior clinical efficacy compared to the traditional BT technique for the management of segmental femoral bone defects, with shorter time in frame, lower external fixation index, and reduced complications. Therefore, the SRN technique may be considered an optimal choice for treating patients with such conditions.

## Introduction

Segmental femoral bone loss resulting from open fractures, osteomyelitis, or nonunion present’s significant challenges for trauma surgeons, leading to acute and long-term morbidity for patients^1^. Various approaches have been employed to manage this condition, including Ilizarov bone transport (BT), staged bone transplantation with membrane-induced procedures, and fibular transplantation^1–6^. The Ilizarov BT technique is widely utilized for limb lengthening, deformity correction, and reconstruction of segmental bone loss. This treatment mainly involves two stages: distraction osteogenesis and consolidation, with the consolidation phase typically lasting 2–3 times longer than the distraction phase^3^. Both distraction and consolidation are commonly performed using external fixation. However, the prolonged duration of external fixation is associated with a high incidence of frame-related complications and patient discomfort, leading to an extended period for patients to resume normal social and work activities.

Several modified techniques, such as transport/lengthening over a nail, shortening and re-lengthening, transport and then nailing, and multi-focal BT, have been developed^7–13^. The transport/lengthening over a nail technique reduces the time spent in an external frame, thereby significantly decreasing the occurrence of frame-related complications. However, during the distraction osteogenesis period, where both intramedullary nails and external fixators are utilized simultaneously, the interconnection between internal and external fixation may provide a pathway for bacteria to enter the medullary cavity through pin tracts, potentially causing deep infections^7,13^, which may be catastrophic for these patients. Although the shortening and re-lengthening technique can also reduce the external fixation index, external fixation is still required during both the re-lengthening and consolidation periods, resulting in a long time in frame^8^. Several scholars have demonstrated that the technique of first BT followed by nailing is a good option for patients with segmental bone defects^10,11^. However, this technique often necessitates bone grafting, which additionally increases the patient’s trauma.

The authors have adopted shortening/re-lengthening and nailing (SRN) technique as a means of addressing the challenges associated with the treatment of patients afflicted with segmental femoral bone defects. This approach aimed to overcome the shortcomings of traditional Ilizarov BT technique, as well as various modified methods, including the lengthening over a nail technique. Patients with substantial femoral bone defects initially underwent acute shortening of the affected limb, and then were lengthened by a monorail external fixator. Upon achieving equivalent length to the uninjured limb, the external fixator was removed. Subsequently, an intramedullary nail was inserted across the regenerated bone, remaining in place until the consolidation period was completed. Notably, unlike the lengthening over a nail technique, the application of both internal fixation and external fixators did not occur simultaneously within the injured limb for the SRN technique. This distinctive aspect served to alleviate concerns among surgeons and patients regarding the potential recurrence of deep infections. The primary objective of this study was to present the authors' experiences and outcomes associated with the implementation of the SRN technique for the treatment of segmental femoral bone defects.

## Materials and methods

### Patients

This study was approved by the Biomedical Research Ethics Committee of Hong Hui Hospital and adhered to relevant guidelines and regulations. A retrospective analysis was conducted by the authors. The clinical and radiological data of 46 cases with segmental femoral bone defects treated by the authors between June 2014 and June 2020 were included. Inclusion criteria were as follows: (i) patients diagnosed with post-traumatic segmental femoral bone defects with a bone defect length greater than 3 cm; (ii) patients with post-traumatic femoral bone defects attributed to acute trauma or post-traumatic osteomyelitis; (iii) patients managed by the SRN or Ilizarov BT methods; (iv) patients with complete medical records. Exclusion criteria were defined as follows: (i) segmental femoral bone defects resulting from malignant tumors or congenital diseases; (ii) patients with severe comorbidities unable to tolerate anesthesia or surgery; (iii) patients lost to follow-up.

### Preoperative management

In order to assess infection-related markers, routine blood sampling tests were conducted. Wound secretions were examined for bacterial culture and the presence of antibiotic resistance genes. Routine preoperative evaluations included X-ray and CT scans of the injured limb. In cases of osteomyelitis, magnetic resonance imaging (MRI) was employed to determine the extent of infection. Radionuclide examinations were conducted to detect any intramedullary jump lesions.

### Operation protocol of SRN group

The initial step in the SRN technique involved the implementation of shortening. Following radical debridement, segmental femoral bone defects would occur, which could be addressed by acutely shortening the injured limb. In cases where complete acute shortening could not be achieved in one instance, the femur underwent a combination of acute and gradual shortening until the defect site was temporarily eliminated. Simultaneously, the docking site was temporarily secured.

Re-lengthening was undertaken once the soft tissues had healed. The temporary fixation device was removed, and a monorail lengthening frame was installed. Using a thin wire saw and performing low-energy osteotomy beneath the periosteum, the osteotomy plane was situated at the metaphysis. The initial rate of lengthening was set at 1.0 mm per day, with adjustments made based on callus formation and the patient's tolerance. Lengthening continued until both extremities attained equal length, at which point the lengthening frame was removed.

**Figure 1:**
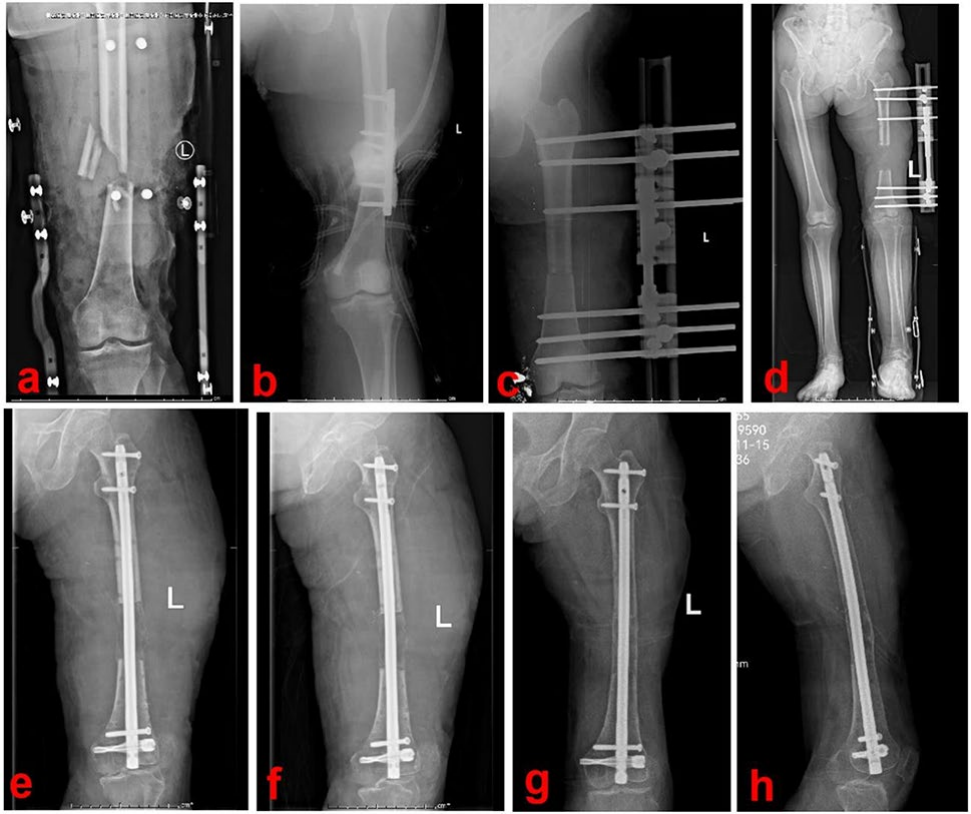
A 58-year-old female was successfully treated by the SRN technique. (**a**) and (**b**): The patient suffered from a severe open fracture and was acutely shortened; (**c**) and (**d**): After soft tissues healed, re-lengthening was performed via a monorail external frame; (**e**) and (**f**): When the limb length was restored the external frame was removed and an intramedullary nail was inserted; (**g**) and (**h**): Six months after removing the external frame, the consolidation of the new callus was good. SRN: shortening/re-lengthening and nailing.

Subsequently, a femoral intramedullary nail was inserted following routine procedures. An antegrade or retrograde intramedullary nail was selected according to the location of the lengthening segment. The proximal and distal ends of the intramedullary nail were locked to prevent limb shortening. Throughout the entire consolidation stage, the intramedullary nail was used to maintain femoral stability. A typical case of the SRN group is shown in Fig. 1.

### Operation protocol of BT group

**Figure 2:**
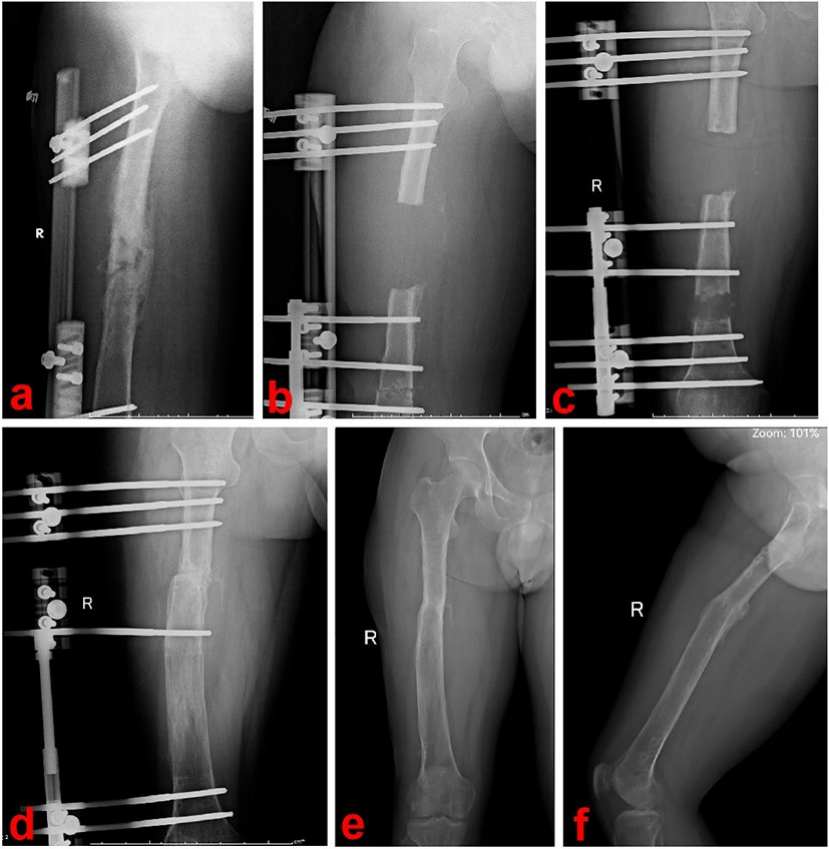
A 29-year-old male was treated by the traditional BT technique. (**a**) The patient suffered from osteomyelitis of middle femur; (**b**–**d**): the BT technique was performed after segmental resection; (**e**) and (**f**): after consolidation of new callus was completed, the monorail external frame was removed. *BT* bone transport.

In the BT group, a monorail external frame was employed to ensure stability throughout the distraction osteogenesis and consolidation stages. The length of the injured limb was adjusted to match that of the unaffected limb during the transport phase. Once callus consolidation was achieved, the monorail lengthening frame was removed. Figure 2 depicts a representative case from the BT group.

### Postoperative management

Post-surgery, infection-related markers were assessed, and a six-week course of systemic antibiotics was administered via intravenous drip or oral administration. Patients were encouraged to engage in limb functional exercises and partial weight-bearing. Over the subsequent months, follow-up appointments were scheduled every two weeks. Outpatient review was preferred. Other methods included telephone, Internet messages, and WeChat platforms. Key aspects of follow-up included evaluating callus formation, monitoring for complications, and assessing limb tolerance. In the event of complications, prompt treatment was administered through non-surgical or surgical approaches. Pin-tract care was conducted once daily following surgery.

### Outcome indexes

Outcome indexes included the time spent in the monorail frame, the external fixation index, SAS scores^14^, bone healing and functional results. The time in frame referred to the total duration from the installation of the transport/lengthening frame until its removal. The external fixation index was calculated by dividing the time in frame by the length of the femoral bone defects. SAS scores were determined through a subjective test scale administered one month after the installation of the transport/lengthening frame. Bone healing outcomes were evaluated using the Paley score, classified as excellent, good, fair, or poor^15^. Knee joint functions were assessed using the Hospital for Special Surgery (HSS) score^16^, while hip joint functions were evaluated using the Harris score^17^. Postoperative complications were classified according to the Paley criteria^18^, which included problems, obstacles, and sequelae. Problems referred to complications that could be addressed through non-surgical methods, while obstacles required surgical intervention. Sequelae denoted complications that remained unresolved.

### Statistical methods

The data were analyzed using SPSS 24.0 software (IBM Co., USA). Measurement data were presented as mean ± standard deviation and compared utilizing an unpaired *t* test, including variables such as age, bone defect length, follow-up duration, time in frame, external fixation index, HSS score, and Harris score. Count data were presented as numbers or numbers with incidence, and analyzed using the χ^2^ test. Count data included variables such as gender, etiology, affected side, bone healing score, SAS score, and complications. A *p* value of less than 0.05 was considered statistically significant.

### Consent to participate/consent to publish

All patients or their family members have signed the informed consent before surgery and provided the consent to publish and report individual clinical data.

## Results

General data are shown in Table 1. In this study, a total of 46 patients diagnosed with segmental femoral bone defects were included, consisting of 34 males and 12 females. The mean age was 35 ± 8 years in the SRN group and 33 ± 9 years in the BT group. The mean bone defect lengths were 5.8 ± 1.1 cm for SRN patients and 6.1 ± 1.6 cm for BT patients. The average follow-up periods were 30 ± 6 months for SRN and 29 ± 5 months for BT groups. There were 11 patients suffering from acute trauma and 10 with osteomyelitis in the SRN group; there were 9 patients suffering from acute trauma and 16 with osteomyelitis in the BT group. SRN group had 9 left-sided and 12 right-sided cases; BT group had 14 left-sided and 11 right-sided cases. No significant differences were observed in the general data between the two groups (*p* > 0.05).

**Table 1:** Demographics of the two groups.

Variable	SRN group (n = 21)	BT group (n = 25)	*p* value
Age (year)	35 ± 8	33 ± 9	0.430
Gender (M/F)	16/5	18/7	0.747
Etiology			0.264
Acute trauma	11	9	
Osteomyelitis	10	16	
Side (L/R)	9/12	14/11	0.374
Bone defect length (cm)	5.8 ± 1.1	6.1 ± 1.6	0.458
Follow-up (month)	30 ± 6	29 ± 5	0.547

*SRN* shortening/re-lengthening and nailing, *BT* bone transport.

Table 2 presents the clinical results of the SRN and BT groups. The mean time spent in the frame was 3.7 ± 1.4 months for SRN patients and 9.4 ± 3.7 months for BT patients, with a significant difference (*p* < 0.05). The external fixation index was 0.62 ± 0.12 months/cm for SRN patients and 1.50 ± 0.19 months/cm for BT patients, and the difference was also significant (*p* < 0.05). The docking site achieved firm healing in all patients. Regarding bone healing, no significant difference was observed between the SRN and BT groups (*p* = 0.237). In the SRN group, 18 cases achieved excellent recovery, two had good recovery, and one showed fair recovery. Similarly, in the BT group, 16 patients had excellent recovery, five had good recovery, and 4 had fair recovery. Analysis of self-rating anxiety scale data revealed that in the SRN group, 16 patients were not troubled with anxiety, three experienced mild anxiety, and two had moderate anxiety. However, in the BT group, five patients were not troubled with anxiety, 12 had mild anxiety, and eight suffered from moderate anxiety. In summary, SRN patients experienced significantly less anxiety compared to BT patients (*p* < 0.05). The mean HSS score was 87 ± 6 points for SRN patients and 76 ± 8 points for BT patients, with a significant difference (*p* < 0.05). However, there were no significant differences in Harris scores between the SRN and BT groups (*p* = 0.446).

**Table 2:** Clinical evaluation indexes for the two groups.

Variable	SRN group (n = 21)	BT group (n = 25)	*p* value
Time in frame (month)	3.7 ± 1.4	9.4 ± 3.7	0.001
External fixation index (months/cm)	0.62 ± 0.12	1.50 ± 0.19	0.001
SAS score			0.001
No anxiety	16	5	
Mild anxiety	3	12	
Moderate anxiety	2	8	
Bone healing score			0.237
Excellent	18	16	
Good	2	5	
Fair	1	4	
HSS score (points)	87 ± 6	76 ± 8	0.001
Harris score (points)	86 ± 10	84 ± 7	0.446

*SRN* shortening/re-lengthening and nailing, *BT* bone transport, *SAS* self-rating anxiety scale, *HSS* hospital for special surgery.

Table 3 presents the postoperative complications of the SRN and BT groups. The mean number of complications per patient was 0.9 ± 0.7 in the SRN group and 1.8 ± 0.7 in the BT group, with a significant difference (*p* < 0.05). There were no statistically significant differences in the incidence of each specific complication between the SRN and BT groups (*p* > 0.05). However, when combining Grade-II and Grade-III pin-tract infections, the incidence of pin-tract infections in the SRN group (7/21, 33.3%) was significantly lower than that in the BT group (17/25, 68.0%) (*p* < 0.05).

**Table 3:** Complications of the two groups.

Complications	Number of complications (SRN group, n = 21)	Number of complications (BT group, n = 25)	*p* value
Problem			
Grade-II pin-tract infection	4	11	0.072
Transient loss of knee movement	3	5	0.905
Transient loss of hip movement	1	2	0.876
Delayed maturation of regenerate site	1	3	0.732
Obstacles			
Grade-III pin-tract infection	3	6	0.650
Axial deviation	1	4	0.457
Docking site nonunion	2	5	0.566
Soft tissue invagination	0	1	–
Sequelae			
Joint stiffness	1	3	0.732
Varus or valgus deformity	1	2	0.876
Limb inequality	1	2	0.876
Total (number of complications)	18	45	
Number of complications per patient (mean)	0.9 ± 0.7	1.8 ± 0.7	0.001

*SRN* shortening/re-lengthening and nailing, *BT* bone transport.

All “problems” (treated by non-surgical methods) and “obstacles” (treated by surgical methods) were actively addressed for both SRN and BT patients. Patients with Grade-II pin-tract infections received antibiotics and dressing. To improve transient loss of joint movement, active functional exercises and manual release under oral painkillers were performed. Prolonging the time spent in the frame was used to treat patients with delayed maturation of the regenerate site. For those with Grade-III pin-tract infections, the affected pin was removed and replaced. Axial deviation was corrected through additional surgery. Docking site nonunion was treated with autogenous bone grafting, and soft tissue invagination was resolved through surgical release. However, patients with "sequelae" declined further surgeries.

## Discussion

This study demonstrated that both SRN and BT techniques yield favorable outcomes in terms of bone healing and functional recovery for the management of segmental femoral bone defects caused by acute trauma or posttraumatic osteomyelitis. In our series, all cases achieved successful union at the docking site and consolidation of new callus, effectively preventing the need for amputation. Notably, SRN treatment resulted in superior knee joint function compared to BT treatment. Additionally, patients undergoing SRN experienced lower levels of anxiety. Importantly, the SRN technique showed advantages in terms of shorter time spent in the frame and a lower mean external fixation index compared to the BT technique. These factors likely contributed to the lower incidence of complications observed in SRN treatment compared to BT treatment.

The BT technique is commonly employed for the treatment of large bone defects. However, this technique is associated with several postoperative complications, including pin-tract infections and docking site nonunion. Furthermore, the lengthy consolidation time required for BT can be challenging for patients, particularly those with segmental femoral bone defects. To address these issues, some researchers have explored the use of acute shortening and re-lengthening techniques, which offer advantages over classical BT, such as a higher rate of docking site union, an easier maintenance for alignment, and fewer complications^19,20^. Notably, Sigmund et al. reported a lower rate of docking site surgery using the acute shortening technique compared to BT technique^21^. However, patients still need to wear an external frame during the distraction osteogenesis and consolidation stages, which may necessitate a relatively prolonged time in the frame. Another approach utilized by researchers is BT/lengthening over a nail to treat post-traumatic bone defects. Studies by Lu et al.^22^ and Oh^23^ reported the mean external fixation index to be 23.88 days/cm and 26 days/cm, respectively, using a single-armed external frame over a nail for BT/lengthening. Oedekoven et al. indicated that the external fixation index was 19.42 days/cm for tibial defects and 15.93 days/cm for femoral defects when employing BT over a nail^24^. However, the combined use of external and internal devices raises concerns about the recurrence of deep infections. Several studies have reported deep infection incidences ranging from 2.4 to 15% with the use of transport/lengthening over a nail technique^22,25–27^, although very few patients required amputation due to severe deep infection^28^.

Our research team has been working on the SRN method for the management of segmental femoral bone defects over the course of several years. Based on our data, we found that the external fixation index in patients undergoing the SRN method was 0.62 ± 0.12 months/cm. These results were comparable to previous studies on BT over a nail. Importantly, in our study, patients treated with the SRN technique did not experience any recurrence of deep infections. It was achieved by dividing the reconstruction procedure into two distinct phases, separating the internal fixation phase from the external fixation phase. This approach helped minimize the occurrence of deep infections. Upon removal of the lengthening frame, patients felt comfortable and highly satisfied. In fact, the SAS scores were significantly lower for SRN patients compared to BT patients, indicating a notable release of anxiety after removing the lengthening frame. Furthermore, our study revealed that the mean number of complications per patient was lower in the SRN group compared to the BT group. This can be attributed to the early removal of the lengthening frame in the SRN group. Specifically, seven cases in the SRN group experienced pin-tract infections, while this number rose to 17 in the BT group. This difference may be attributed to the significantly different duration of time spent in the lengthening/transport frame for SRN and BT patients. Consequently, our SRN method proved to be more effective in preventing pin-tract infections and reducing the need for additional surgeries.

In a study by Rozbruch et al., a retrospective case-matched comparison was conducted between the lengthening and then nailing technique and the classic lengthening technique^29^. They found that the lengthening and then nailing technique offered advantages over the classic method, including shorter external fixation times, faster bone healing, and protection against re-fracture. However, in their study, the lengthening and then nailing technique was performed using an annular Ilizarov/Taylor Spatial Frame in 39 limbs (35 tibias and four femurs) of 27 selected patients. This frame is tolerable for the tibia, but challenging for the femur. In our study, a single-armed external frame was used for patients with femoral bone defects, which offers greater comfort during the distraction phase compared to an annular frame. Rozbruch’s study also mentioned that 24 of the limbs underwent lengthening and then nailing surgery due to short stature. Although there were no reports of neurovascular traction injuries or chronic pain, these issues are major concerns for such patients. In another study by Sen et al., a combined technique called short supracondylar nail-augmented acute shortening/lengthening was utilized for the treatment of infected nonunion of the distal femur with bone loss^30^. This study demonstrated that the combined strategy was effective in treating distal femoral segmental bone defects following debridement of osteomyelitis, with a high union rate and acceptable complication rates. The mean external fixation index in Sen's study was 29.2 days/cm, which is similar to our results. In our study, we treated bone defects in the proximal, middle, and distal ends of the femur using full-length femoral intramedullary nails for sequential treatment. Full-length intramedullary nails offer greater stability compared to short intramedullary nails, which facilitates early weight bearing and improves patient outcomes.

However, there were several limitations in our research. One key limitation was that it was a retrospective study. The lack of random assignment may have introduced selection bias, as the chief surgeon may have had preferences when deciding on the surgical plan. This potential bias could have an unknown impact on the conclusions of this study. Additionally, the limited number of cases in our study should be considered when interpreting the bone and functional results. It would be beneficial to conduct a longer follow-up period to assess the long-term clinical effects of the SRN and BT techniques. Furthermore, a multi-center prospective study with a larger sample size is warranted to overcome the limitations and shortcomings of this study.

## Conclusion

The SRN technique has shown advantages over the BT technique in terms of reducing the external fixation index and postoperative complications. Patients with segmental femoral bone defects have reported high levels of satisfaction when treated with this technique. In summary, the SRN technique is an effective method for managing segmental femoral bone defects.

## Data Availability

The datasets analyzed during the current study are available from the corresponding author upon reasonable request.
